# Attributable mortality of *Acinetobacter baumannii *infections in critically ill patients: a systematic review of matched cohort and case-control studies

**DOI:** 10.1186/cc4869

**Published:** 2006-03-21

**Authors:** Matthew E Falagas, Ioannis A Bliziotis, Ilias I Siempos

**Affiliations:** 1 Alfa Institute of Biomedical Sciences (AIBS), 9 Neapoleos Street, 151 23 Marousi, Greece; 2 Department of Medicine, Tufts University School of Medicine, 136 Harrison Avenue, Boston, MA 02111, USA

## Abstract

**Introduction:**

There has been a continuing controversy about whether infection with *Acinetobacter baumannii *increases morbidity and mortality independently of the effect of other confounding factors.

**Methods:**

We performed a systematic review of matched case-control and cohort studies examining the mortality attributable to infection with or acquisition of *A. baumannii *(infection or colonization). We included in our review studies that compared mortality and/or morbidity of patients with acquisition of or infection with *A. baumannii *(cases) with the outcomes of matched patients without *A. baumannii *isolation from clinical specimens (controls). The relevant studies were identified from searches of the PubMed and the Cochrane Library databases. Two independent reviewers performed the literature search, study selection, and data extraction from nine identified relevant studies.

**Results:**

The attributable mortalities, in the hospital and in the intensive care unit, of patients with *A. baumannii *infection in six matched case-control studies included in our review ranged from 7.8% to 23% and from 10% to 43%, respectively. In addition, a statistically significantly higher mortality was reported for patients with *A. baumannii *acquisition; that is, colonization or infection (cases) compared with controls without such an acquisition in all four reviewed studies that reported data on this comparison.

**Conclusion:**

Although definitive statements about the mortality attributable to the acquisition of *A. baumannii *cannot be made from the available studies because of their methodological heterogeneity, the reviewed data suggest that infection with or acquisition of *A. baumannii *seems to be associated with increased mortality.

## Introduction

*Acinetobacter baumannii *is a ubiquitous, non-fermenting, aerobic Gram-negative bacterium with intrinsic resistance to multiple antimicrobial agents [1,2]. During the past few decades the organism has emerged as an important nosocomial pathogen, affecting mainly severely ill patients in the intensive care unit (ICU) setting worldwide. *A. baumannii *has been recognized as a leading cause of nosocomial pneumonia and bacteremia (related to central venous catheters or not) in several hospitals in various parts of the world [3-6].

However, there has been a continuing controversy over whether colonization - and, even more importantly, infection – with *A. baumannii *increase morbidity and mortality independently of the effect of other confounding factors. Although several investigators provided evidence that *A. baumannii *infections may be associated with considerable mortality [7-10], some of them support the possibility that the clinical course of critically ill patients may be influenced by many variables and that subsequently the acquisition of or infection with *A. baumannii *may not independently lead to poorer outcomes [11-13]. This controversy has caused considerable confusion among clinicians and investigators about the mortality associated with of *A. baumannii *infections. We therefore sought to systematically identify and synthesize the available evidence about the mortality attributable to acquisition of or infection with *A. baumannii *in critically ill patients by retrieving the available data from relevant matched case-control studies.

## Materials and methods

### Search strategy

Two independent reviewers (IAB and IIS) performed the literature search, study selection, and data extraction. Any disagreement between the two reviewers was resolved by consensus in meetings of all authors. We searched for studies indexed in the PubMed and Cochrane Library (part of which is also the Cochrane Central Register of Controlled Trials) databases by using the following key terms: '*Acinetobacter*', 'mortality', 'colonization', 'case-control', 'match', 'length of stay', and/or 'ICU'. No limits were set in our literature search about the time or language of publication. The references from the identified articles were also searched for relevant publications.

### Study selection

Studies included in our systematic review were case-control or matched cohort studies that compared mortality and/or morbidity of patients with acquisition of or infection with *A. baumannii *(cases) with the outcomes of matched patients without *A. baumannii *isolation from clinical specimens (controls).

### Data extraction

We extracted data about the date, setting, and patient population from the studies selected. In addition, the site of infection, the numbers of cases and controls, the methodology for the matching of controls to cases, and clinical outcomes of interest were extracted.

### Outcomes

The main outcomes that we examined in our systematic review were the crude ICU and/or in-hospital mortality of cases and controls, as well as the mortality attributable to acquisition of or infection with *A. baumannii*. The mortality attributable to colonization or infection by *A. baumannii *was determined by subtracting the crude mortality of controls from the crude mortality of cases. In addition, the length of stay in the ICU or in the hospital was reviewed as a secondary outcome.

## Results

### Selected studies

**Figure 1:**
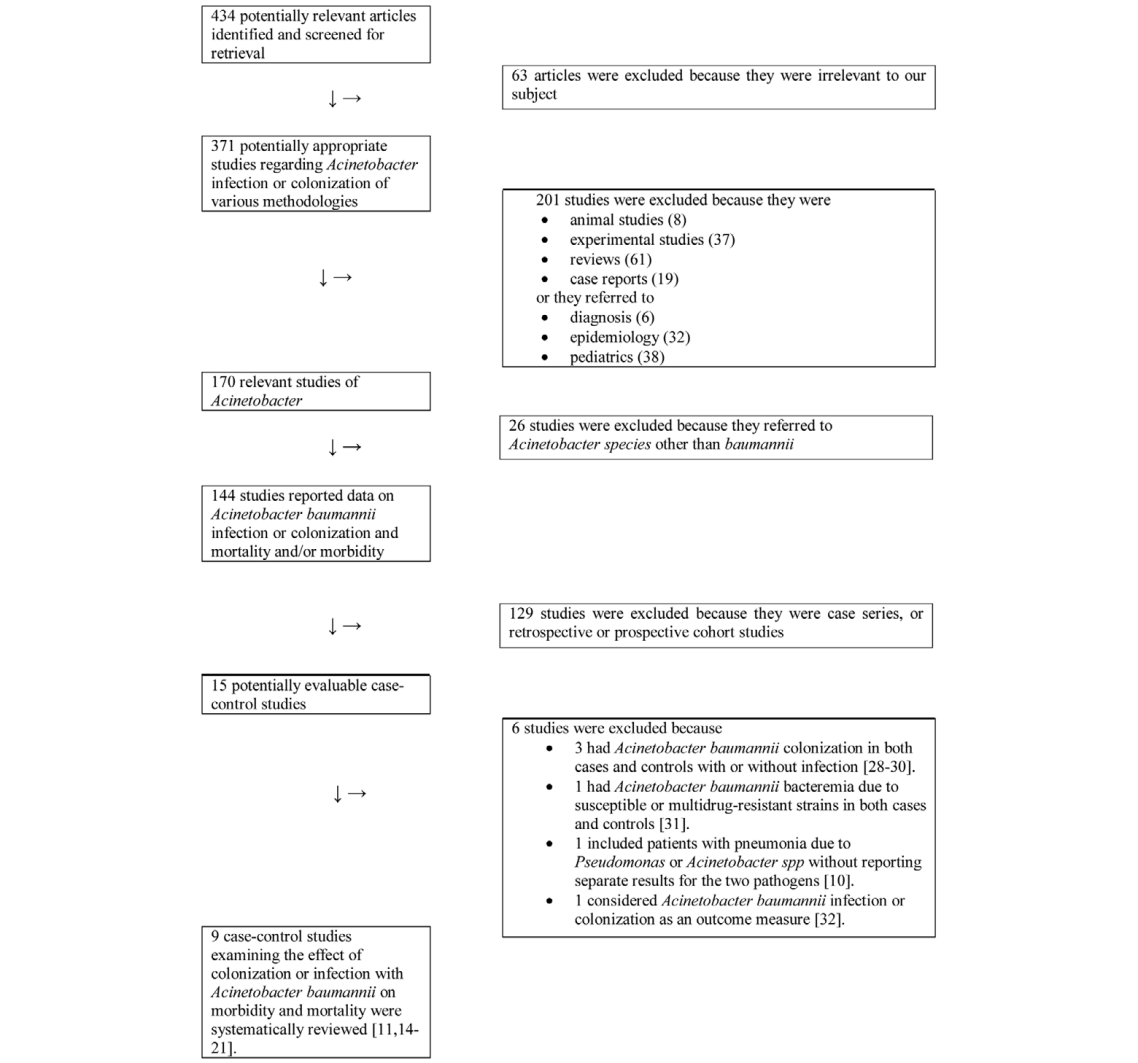
Flow diagram of reviewed articles.

The steps that we followed to select the relevant studies for our analysis are presented in Figure 1. We initially identified 434 potentially relevant studies from the search of the PubMed and Cochrane Library databases as well as from reading the references of relevant studies. In the end there were nine case-control studies (six retrospective [11,14-18], one prospective [19], and two with mixed, bi-directional study design, in which cases were studied prospectively but controls were identified from retrospective data review [20,21]) that compared outcomes in patients colonized or infected with *A. baumannii *(cases) with those of matched patients from whom *A. baumannii *were not isolated [11,14-21].

We present the main characteristics of the analyzed studies, as well as the outcomes of our interest in cases with *A. baumannii *infection and controls, in Table 1. As shown, the infection sites for the cases with *A. baumannii *infection were the lower respiratory tract (ventilator-associated pneumonia (VAP)) in one study [11] and blood (primary or secondary bacteremia) in another two studies [14,18]. In four of the remaining studies both colonization and infection with *A. baumannii *were described, regardless of the affected site. In the two studies that reported on cases with *A. baumannii *infection in the bloodstream, the controls might have been infected with *A. baumannii *but did not have a bloodstream infection with the pathogen. Data on the characteristics of the studies as well as reported outcomes for patients with acquisition (colonization or infection) of *A. baumannii *and outcomes for controls without *A. baumannii *acquisition are presented in Table 2.

### Mortality

Four studies reported data on in-hospital mortality in patients infected with *A. baumannii*, in comparison with controls not infected with the microorganism (Table 1) [11,14,15,18,20,21]. In all four studies there was increased mortality in patients infected with *A. baumannii *in comparison with controls, although the difference was not statistically significant. In one of these studies the mortality difference between the compared groups almost reached statistical significance [18]. The mortality attributable to *A. baumannii *infection in these studies ranged from 7.8% to 23%. In addition, three studies reported data about the mortality of cases and controls in the ICU [11,14,21]. In all three studies mortality in the ICU was higher in patients infected with *A. baumannii *than in controls. In one of these studies the difference in mortality between cases and controls was statistically significant [21]. Attributable mortality in the ICU ranged from 10% to 43% in the reviewed studies.

Four studies reported mortality data in patients with *A. baumannii *acquisition (colonization or infection with *A. baumannii*), in comparison with controls who were not colonized nor infected with *A. baumannii *(Table 1) [16,17,20,21]. In-hospital mortality and mortality in the ICU were reported in three studies [16,17,20] and one study [21] respectively. Interestingly, in all four studies mortality was statistically higher in patients colonized or infected with *A. baumannii *than in controls. The attributable in-hospital mortality of *A. baumannii *infection in the three studies that reported on this outcome ranged from 16% to 27%, whereas in the other study the attributable mortality in the ICU was 30%. It is noteworthy that two of these four studies did not match the patients for disease severity [16,17].

### Length of stay in the ICU

Five out of seven studies that reported data on mortality in patients infected with *A. baumannii *(Table 1) also provided data on the length of stay of cases and controls in the ICU [11,14,17,18,21]. In three of these five studies a statistically significant increase in the length of stay in the ICU was reported for the cases with *A. baumannii *infection [14,17,21], whereas in the remaining two studies no significant difference was found in the length of stay in the ICU between cases and controls.

Data on the length of stay of cases and controls in the ICU was reported in all five studies that examined the effect of acquisition (colonization or infection) of *A. baumannii *(Table 2). A statistically significant increase in the length of ICU stay was noted in four of these five studies for patients who were colonized or infected with *A. baumannii *(cases) in comparison with patients from whom this bacterium was not isolated (controls) [16,17,20,21] (no statistical data on the comparison of this outcome in the studied population were reported in the remaining study [19]).

## Discussion

The attributable mortalities, in hospital and in the ICU, of patients with *A. baumannii *infection in the reviewed matched case-control and cohort studies ranged from 7.8% to 23% and from 10% to 43%, respectively. It should be emphasized that all studies that examined mortality of patients (cases) with *A. baumannii *acquisition (colonization or infection) compared with controls without such an acquisition found statistically significant differences; that is, higher mortality in cases than in controls, although a causative role for the isolate on the mortality cannot be directly inferred from these data. In addition, no matching of patients and controls for disease severity was made in two of these studies [16,17]. Further, the length of stay in the ICU was found to be statistically significantly increased in patients with *A. baumannii *infection in three of five studies examining this outcome.

The increase in mortality of patients with infection or acquisition of *Acinetobacter *in comparison with matched controls without colonization or infection, noted in the studies included in the systematic review, is supported by evidence provided by several retrospective and prospective cohort studies examining this issue. For example, Kollef and colleagues [22] found that VAP due to non-fermentative Gram-negative pathogens was independently associated with increased mortality in hospital, with an associated mortality rate of 65%. In that study the occurrence of late-onset VAP due to non-fermentative Gram-negative pathogens was the most important predictor of hospital mortality in patients developing VAP (adjusted odds ratio 5.4; 95% confidence interval 2.8 to 10.3; *p *= 0.009).

In addition, Garrouste-Orgeas and colleagues [23], in a 1-year prospective observational survey, evaluated the clinical effect of salivary or rectal carriage of multi-resistant *Acinetobacter baumannii *and/ or *Klebsiella pneumoniae *in patients hospitalized in an ICU. Of 265 patients, 88 (33%) developed oropharyngeal and/or rectal carriage. Mortality was significantly greater in the carrier group (43% versus 25%, *p *< 0.001). Stratification of patients showed that, although abnormal carriage was found in the most severely ill patients, it mainly influenced mortality in the less severely ill. Finally, Wisplinghoff and colleagues [24], reported results from the SCOPE (Surveillance and Control of Pathogens of Epidemiologic Importance) project, a prospective study with 49 participating hospitals in the USA. The authors reported that the mortality of patients with 111 bloodstream infections caused by *A. baumannii *was not significantly different from that of 2,952 patients with bloodstream infections due to other Gram-negative pathogens (35/111 patients with *A. baumannii *died (31.5%) compared with 821/2,952 patients with other Gram-negative pathogens (27.9%)). This study provided strong evidence in support of the position that *A. baumannii *bacteremias are as severe as other Gram-negative bacteremias and thus may result in considerable mortality.

Some of the investigators studying patients with *A. baumannii *infections concluded that mortality in these patients was not independently associated with these infections. In two studies by Garnacho-Montero and colleagues [11,25], one of which was included in our review, the authors suggested that VAP due to *A. baumannii *was not associated with a poorer prognosis than other causes of VAP. In addition, Weingarten and colleagues [17], in another study included in our review, suggested that colonization or infection with *A. baumannii *is not associated with increased mortality, but instead that the severity of the illness of cases and controls is the major determinant of mortality. Finally, Sofianou and colleagues [26], in a prospective cohort study examining the incidence, risk factors and pathogens of VAP, concluded that the occurrence of VAP, regardless of the microbiological etiology, was not associated with higher mortality in 198 ICU patients. However, this finding was in disagreement with those from other studies, including that of Fagon and colleagues [10], in which mortality was higher in cases with VAP caused by *A. baumannii *and *P. aeruginosa *than in controls with bronchial colonization with these pathogens.

Although the aforementioned studies attempted to unravel the prognostic importance of colonization or infection with *A. baumannii*, the disagreement between their results create difficulties in deriving definitive conclusions about the severity of disease that results from this organism. The fact that the organism is often resistant to multiple antimicrobial agents, making it difficult to provide appropriate antibiotic therapy, and also the fact that it affects critically ill patients, make the answer of the above question of crucial importance for clinicians worldwide.

Our systematic review has several limitations. First, we selected for inclusion only matched case-control and cohort studies in our attempt to provide data from comparative studies with analytical methodology. However, it should be noted that different matching criteria were used in the studies included in our review and also that some studies did not take into account the severity of disease as a matching criterion. Second, no specific analysis was provided in the reviewed studies about the effect of colonization or infection of *A. baumannii *with various phenotypes (*in vitro *susceptibility pattern to various antibiotics) [27]. Third, we could not pool the data by using the techniques of meta-analysis because there was considerable heterogeneity in the sites of infections, the populations studied, and, most importantly, matching criteria between the studies. However, although no statistically significant differences were found in the comparison of mortality between patients with *Acinetobacter *infection (cases) and controls without such infection it should be noted that this outcome is probably due to the small sample sizes in the studies included in our systematic review. Thus, either more homogeneous data from studies that would allow a meta-analysis or larger studies with enough power could offer a definite answer to our research question. Last, we did not evaluate the effect of *A. baumannii *infection at various body sites and systems such as pneumonia and bacteremia on mortality.

## Conclusion

The evidence from the reviewed matched case-control and cohort studies examining the mortality of patients with colonization or infection with *A. baumannii *suggests that such colonization and infection might be associated with considerably increased mortality. It should be emphasized that to attribute the difference in mortality between cases and controls directly to colonization or infection with *Acinetobacter *(attributable mortality) is more than a simplified approach to this complex issue. This is because the reviewed studies did not and could not match for other factors that might have made important contributions to mortality. Despite these shortcomings, our systematic review lends support to the idea that *A. baumannii *infections are associated with considerable morbidity and mortality, and clinicians should therefore make every effort to combat them.

## Key messages

• 	There has been controversy over whether colonization and infection with *A. baumannii *increase morbidity and mortality independently of other factors.

• 	The attributable mortality, in hospital and in the ICU, of patients with *A. baumannii *infection in six matched case-control studies included in our review ranged from 7.8% to 23% and from 10% to 43%, respectively.

• 	Statistically significantly higher mortality was reported for patients with *A. baumannii *acquisition; that is, colonization or infection (cases) compared with controls without such an acquisition in all four reviewed studies that reported data on this comparison.

• 	Definitive statements cannot be made about the attributable mortality of acquisition of *A. baumannii *from the analysis of the results from the reviewed studies because of their methodological heterogeneity.

## Abbreviations

ICU = intensive care unit; VAP = ventilator-associated pneumonia.

## Competing interests

The authors declare that they have no competing interests.

## Authors' contributions

MEF had the idea, designed and supervised the study, and is the guarantor. IIS and IAB performed the literature search, identified the relevant studies to be included in the analysis, and extracted the data for the study. MEF and IAB wrote a first version of the manuscript. All authors made substantial revisions of the manuscript and approved its final version.

## Figures and Tables

**Table 1:** Characteristics and outcomes of matched cases and controls (patients infected and non-infected with *A. baumannii*)

Reference	Site of infection; patients and setting	Cases	Controls	Matching of controls to cases	Mortality					Length of ICU stay (days)	
					Cases	Controls	*p*	Attributable mortality (%)	Cases	Controls	*p*

[14]	Bacteremia; medical, surgical, burn, and cardiac surgery ICUs of a hospital in Belgium	45 patients with *Ab *bacteremia	90 patients without bacteremia (excluding coagulase-negative *Staphylococci*)	(1) APACHE II score; (2) primary diagnosis of ICU admission; (3) LOS in the ICU at least as long as that of the case prior to isolation of *Ab*	Hospital: 19/45 (42.2%)	Hospital: 31/90 (34.4%)	0.378	7.8 (95% CI - 9.7 to 25.3)	Mean 28, SD 19.9, median 25, IQR 16–34	Mean 23, SD 20.2, median 20, IQR 8–31	0.043
					ICU: 14/45 (31.3%)	ICU: 19/90 (21.3%)	0.203	10			
[18]	Bloodstream infection; burn ICU in Germany	29 patients with nosocomial *Ab *bloodstream infection	58 matched controls without *Ab *BSI	(1) Date of admission; (2) age; (3) LOS in the ICU at least as long as that of the case before isolation of *Ab*; (4) same unit	Hospital: 9/29 (31%)	Hospital: 8/58 (14%)	0.056	17	Mean 50, SD 27	Mean 30, SD 23	NR
[11]	Microbiologically documented VAP; 4 Spanish ICUs	60 patients with VAP due to *Ab*	60 patients with any documented non-*Ab *infection or no infection at all	(1) Equal or longer duration of stay in ICU before pneumonia; (2) APACHE II score; (3) primary diagnosis of ICU admission	ICU: 24/60 (40%)	ICU: 17/60 (28.3%)	0.17	11.7	Mean 35.3, SD 23.8	Mean 36.6, SD 35.7	NS
[15]	Nosocomial outbreak of resistant *Ab*; MICU in USA	14 patients with *Ab *nosocomial pneumonia and/or bloodstream infection	29 patients mechanically ventilated for at least 7 days without developing *Ab *infection or colonization	(1) Date of admission; (2) mechanical ventilation in the MICU for ≥ 1 week	Hospital: 6/14 (43%)	Hospital: 11/29 (32%)	0.9	11	NR	NR	NR
[21]	Acquisition of *Ab *in critically ill patients in ICU; medical and surgical ICU in Spain	75 patients (48 infected, 27 colonized) with *Ab *isolation	75 patients without any *Ab *isolation	(1) Age; (2) sex; (3) APACHE II; (4) date of admission; (5) primary diagnosis of ICU admission; (6) LOS in the ICU at least as long as that of the case before isolation of *Ab*; (7) Mechanical ventilation for >24 h	ICU: 28/48 (58%)	ICU: 7/48 (15%)	< 0.001	43 (95% CI 34–52)	Mean 30.1, SD 27.2, median 23, IQR 11.5–37	Mean 15.5, SD 19.3, median 10, IQR 7.5–15	< 0.001
[20]	Nosocomial acquisition of MDR *Ab*; MICU in France	40 patients (13 infected and 27 colonized) with *Ab*	40 patients non-infected and non-colonized with *Ab*	(1) APACHE II score; (2) LOS in the ICU at least as long as that of the case before isolation of *Ab*; (3) age; (4) date of admission	Hospital: 7/13 (53.8%)	Hospital: 4/13 (30.8%)	0.23	23	Mean 23.8, SD 9.6	NR	NR
[17]	Colonization or infection with (89% MDR) *Ab*; trauma centre in USA. Most patients were in ICUs	102 patients (33 infected, 69 colonized) with *Ab*	102 controls without *Ab*	(1) Primary diagnosis (computerized codes); (2) same period	Hospital: 14/33 (42%)	NR	NA	NA	Mean 51.33, SD 6.79	Mean 19.00, SD 5.90	< 0.001

*Ab*, *Acinetobacter baumannii*; APACHE, Acute Physiology and Chronic Health Evaluation; CI, confidence interval; ICU, intensive care unit;

IQR, interquartile range; LOS, length of stay; MDR, multidrug resistant; MICU, medical ICU; NA, non-applicable; NR, not reported; NS, non-significant; VAP, ventilator-associated pneumonia.

**Table 2:** Comparison of patients with acquisition of *A. baumannii *with matched controls

Reference	Site of infection; patients and setting	Cases	Controls	Matching of controls to cases	Colonization or infection						
					Mortality					Length of ICU stay (days)	

					Cases	Controls	*p*	Attributable mortality (%)	Cases	Controls	*p*

[21]	Acquisition of *Ab *in critically ill patients in ICU; medical and surgical ICU in Spain	75 patients (48 infected and 27 colonized) with *Ab*	75 patients without any *Ab *isolation	(1) Age (± 6 years); (2) sex; (3) APACHE II; (4) date of admission; (5) primary diagnosis of ICU admission; (6) LOS in the ICU at least as long as that of the case before isolation of *Ab*; (7) Mechanical ventilation for >24 h	ICU: 37/75 (49%)	ICU: 14/75 (19%)	< 0.001	30 (95% CI 23–37)	Mean 30.7, SD 26.9, median 23, IQR 11–37	Mean 13.8, SD 16.4, median 10, IQR 6–15	< 0.001
[20]	Nosocomial acquisition of MDR *Ab*; MICU in France	40 patients (13 infected and 27 colonized) with *Ab*	40 patients without any *Ab *isolation	(1) APACHE II score; (2) LOS in the ICU at least as long as that of the case before isolation of *Ab*; (3) age; (4) date of admission	Hospital: 20/40 (50%)	Hospital: 10/40 (25%)	0.046	25	Mean 22.6, SD 9.6, median 19, IQR 5–82	Mean 12.3, SD 12.9, median 11, IQR 3–35	< 0.001
[16]	Nosocomial outbreak of MDR *Ab*; ICU in USA	25 patients (9 infected, 15 colonized) with *Ab*	32 patients with cultures negative for *Ab*	(1) Same ICU; (2) date of admission	Hospital: 13/25 (52%)	Hospital: 8/32 (25%)	0.036	27	Mean 19.6, median 18	Mean 6.1, median 4.5	< 0.05
[17]	Colonization or infection due to (89% MDR) *Ab*; trauma centre in USA. Most patients were in medical, surgical, and burns ICUs	102 patients (33 infected, 69 colonized) with *Ab*	102 controls without *Ab*	(1) Primary diagnosis (computerized codes); (2) same period	Hospital: 35/102 (34%)	Hospital: 18/102 (18%)	0.007	16	Mean 27.35, SD 28.21	Mean 5.53, SD 15.87	< 0.001
[19]	Colonization or infection due to *Ab*; university hospital in France. 52% of the patients were hospitalized in an ICU	121 patients (infected or colonized) with *Ab*	121 patients with the same specimen as cases found negative for *Ab*	(1) Same unit; (2) same period; (3) same type of specimen	NR	NR	NR	NR	Mean 29, SD 20	Mean 13, SD 10	NR

Acquisition of *A. baumannii *is defined as patients colonized or infected with the organism; matched controls were those without acquisition of the organism. *Ab*, *Acinetobacter baumannii*; APACHE, Acute Physiology and Chronic Health Evaluation; CI, confidence interval; ICU, intensive care unit; IQR, interquartile range; LOS, length of stay; MDR, multidrug resistant; NR, not reported.
